# Methanolic Extracts from Brown Seaweeds *Dictyota cilliolata* and *Dictyota menstrualis* Induce Apoptosis in Human Cervical Adenocarcinoma HeLa Cells

**DOI:** 10.3390/molecules20046573

**Published:** 2015-04-13

**Authors:** Dayanne Lopes Gomes, Cinthia Beatrice Silva Telles, Mariana Santana Santos Pereira Costa, Jailma Almeida-Lima, Leandro Silva Costa, Tatjana Souza Lima Keesen, Hugo Alexandre Oliveira Rocha

**Affiliations:** 1 Laboratório de Biotecnologia de Polímeros Naturais (BIOPOL), Departamento de Bioquímica, Centro de Biociências, Universidade Federal do Rio Grande do Norte (UFRN), Natal, Rio Grande do Norte-RN 59078-970, Brazil; E-Mails: dayanne_gomes@hotmail.com (D.L.G.); cinthiatelles@yahoo.com.br (C.B.S.T.); marispc_bio@yahoo.com.br (M.S.S.P.C.); biolottus23@yahoo.com.br (J.A.-L.); leandro-silva-costa@hotmail.com (L.S.C.); 2 Programa dePós-graduação em Ciências da Saúde, Universidade Federal do Rio Grande do Norte (UFRN), Natal, Rio Grande do Norte-RN 59078-970, Brazil; 3 Intituto Federal de Educação, Ciência e Tecnologia do Rio Grande do Norte (IFRN), Macau, Rio Grande do Norte-RN 59500-000, Brazil; 4 Intituto Federal de Educação, Ciência e Tecnologia do Rio Grande do Norte (IFRN), Santa Cruz, Rio Grande do Norte-RN 59200-000, Brazil; 5 Laboratório de Imunologia das Doenças Infecciosas, Departamento de Biologia Celular e Molecular, Universidade Federal da Paraíba (UFPB), João Pessoa-PB 58051-900, Brazil; E-Mail: tat.keesen@gmail.com

**Keywords:** tropical seaweeds, methanol extracts, cytotoxicity, HeLa cells

## Abstract

Carcinoma of the uterine cervix is the second most common female tumor worldwide, surpassed only by breast cancer. Natural products from seaweeds evidencing apoptotic activity have attracted a great deal of attention as new leads for alternative and complementary preventive or therapeutic anticancer agents. Here, methanol extracts from 13 species of tropical seaweeds (Rhodophytas, Phaeophyta and Chlorophyta) collected from the Northeast of Brazil were assessed as apoptosis-inducing agents on human cervical adenocarcinoma (HeLa). All extracts showed different levels of cytotoxicity against HeLa cells; the most potent were obtained from the brown alga *Dictyota cilliolata* (MEDC) and *Dictyota menstrualis* (MEDM). In addition, MEDC and MEDM also inhibits SiHa (cervix carcinoma) cell proliferation. Studies with these two extracts using flow cytometry and fluorescence microscopy showed that HeLa cells exposed to MEDM and MEDC exhibit morphological and biochemical changes that characterize apoptosis as shown by loss of cell viability, chromatin condensation, phosphatidylserine externalization, and sub-G1 cell cycle phase accumulation, also MEDC induces cell cycle arrest in cell cycle phase S. Moreover, the activation of caspases 3 and 9 by these extracts suggests a mitochondria-dependent apoptosis route. However, other routes cannot be ruled out. Together, these results point out the methanol extracts of the brown algae *D. mentrualis* and *D. cilliolata* as potential sources of molecules with antitumor activity.

## 1. Introduction

Carcinoma of the uterine cervix is the second most common female tumor worldwide, surpassed only by breast cancer. Its incidence is disproportionately high (>80%) in the developing world and its treatment of disease is usually palliative, aimed only at symptom control [1]. Cancer prevention is the most cost-effective effort for cancer control. Chemoprevention is a strategy to inhibit, delay or reverse human carcinogenesis, using especially naturally occurring mediators [2]. There are many anticancer pathways, such as leading tumor cell apoptosis, the impact of the nucleic acid biosynthesis, induction of DNA structure damage, inhibition of RNA synthesis, prevention of the transcript process, or the impact of protein synthesis and function. From the perspective of cell biology, compound-induced tumor cell apoptosis [3] and inhibition of proliferation [4] might play important roles in the control and prevention of cancer.

Natural products evidencing apoptotic activity have attracted a great deal of attention as new leads for anticancer alternative and complementary preventive or therapeutic agents [5]. Nowadays, seaweed-related products are used widely, not only as health foods, but also in clinical drugs for the prevention and treatment cancer. The protective effects of dietary component in Asian communities, kelps and other red and green seaweed against mammary [6], skin [7] and intestinal cancer [8] are supported by epidemiological data [9] and rodent model studies [10].

Previous results showed that the administration of seaweed powder or extract reduced the incidence rate of tumor cell proliferation in *in vitro* [10,11,12] and *in vivo* animal models [13]. In the 1980s, the development of new screening technologies facilitated the search for new anticancer agents in plants and other organisms, focusing on the tropical and sub-tropical regions of the world [14]. Brazil possesses the largest diversity of seaweeds species in the world, and most of these are found in Northeastern Brazil [15]. Despite this great biodiversity, Northeastern Brazilian seaweeds are relatively underexploited with regard to discoveries of active biological substances.

In view of the great biological diversity of cancer, the combination of different types of therapies used for the treatment of cancer and the search for new substances with antitumor activity have emerged with the prospect of achieving a wide therapeutic efficacy. In this regard, we screened thirteen tropical seaweeds to show their effective antiproliferative activities, and select the most active extracts to detail the corresponding mechanism(s) of action for inducing cell death for further potential application as sources of novel drugs for antitumor therapy.

## 2. Results and Discussion

### 2.1. Cytotoxicity Effect

**Figure 1:**
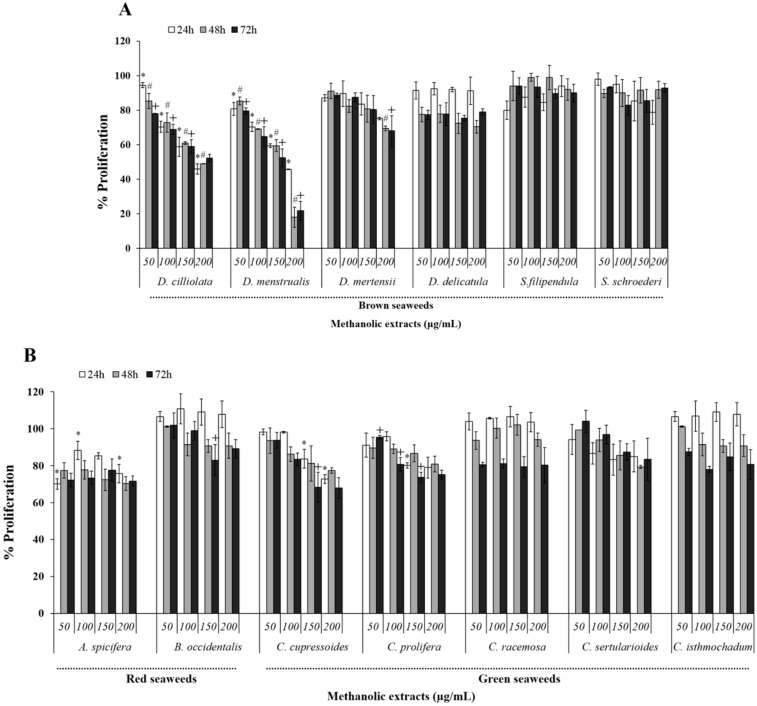
Effect of ME from tropical seaweedson HeLa cells viability after 24, 48 or 72 h of incubation. (**A**) Brown seaweeds and (**B**) red and green seaweeds. Data are expressed as mean ± standard deviation. * Indicates significant difference (*p* < 0.01) among the different concentrations of ME at the time of 24 h. # Indicate significant difference (*p* < 0.01) among the different concentrations of ME at the time of 48 h. + Indicates significant difference (*p* < 0.01) among the different concentrations of ME at the time of 72 h.

In order to analyze the effect of methanolic seaweed extracts (MEs) on uterine tumor cell viability (HeLa) these were cultured with different MEs and their viability was determined using the colorimetric MTT assay. ME of red seaweed promoted a modest inhibition (10% to 20%) of the HeLa cell viability. The dependency of *Botryocladia occidentalis* ME on time and/or dose could not be identified clearly. However, *B. occidentalis* ME presented inhibitory activity of approximately 10% in 24 h that later tended to rise to nearly 20% (Figure 1A,B). With respect to *Acanthophora spicifera* ME, a decreased viability of ~20% was observed already in the lower concentration tested, however this activity did not increase with increasing concentration or time of exposure to the extract.

Although the red seaweed extracts studied here were not effective as antiproliferative agents, other studies show that red seaweed extracts do have this activity. For example, ME (100 µg/mL) of red seaweed *Asparagopsis taxiformis* inhibits about 40% mouse mammary carcinoma cell (EAT) cell proliferation [16]. Another study showed that a ME of *Gloiopeltis furcate* markedly inhibited human hepatocellular carcinoma (HepG2) cell proliferation and induced the G2/M arrest of the cell cycle in a dose-dependent manner (from 10 to 500 µg/mL) [10]. In addition, ME of *Gracilaria corticata* was used against HepG2 and human breast adenocarcinoma (MCF-7) cells. The average inhibitory activity was 91% and 93%, respectively, using 500 µg/mL of extract [17]. However, despite such data, we did not find any studies that have identified the compounds responsible for the antiproliferative action of these red seaweed MEs.

ME of green algae were also able to decrease the rate of HeLa cell viability. However, these inhibitions did not surpass the value of 35% under any of the evaluated conditions. Moreover, the inhibition pattern differed among extracts. The MEs of *Caulerpa prolifera* and *Caulerpa sertularioides* seaweeds showed inhibitory effects under most of the conditions tested. However, in many cases, this effect presented no considerable difference in time and concentration. Nevertheless, they showed a dose-dependent and time-dependent inhibitory activity tendency. ME of *Caulerpa racemosa*, after 24 and 48 h treatment, produced no inhibitory activity, but after 72 h exposure, the average inhibitory activity was 20%, however, this effect was not dose-dependent. *Codium isthimocladum* ME, after 72 h treatment, also showed dose-independent inhibitory activity of around 20%, however different from *C. racemosa* ME, which also exhibited inhibitory activity (~22%) after 48 h under the experimental conditions used. *Caulerpa cupressoides* ME displayed the highest inhibition of HeLa cells, approximately 32%. However, like the two previous MEs it also had just a time-dependent effect.

Another study with MEs of *C. racemosa*, *Cauler papeltata*, *Caulerpa taxifolia* and *Codium elongatum* showed that they are weak antiproliferative agents [16]. These data suggest that seaweeds from these genera do not synthesize antiproliferative compounds with high activity. However, studies with these seaweeds are scarce and more data are needed to confirm this fact.

MEs of the brown seaweeds *Spatoglossum schröederi* and *Sargassum filipendula* presented no antiproliferative activity under any of the tested conditions. *Dictyota mertensii* ME showed significant activity only after 72 h and at the two highest concentrations evaluated. *Dictyopteris delicatula* ME exhibited an inhibition rate of around 22% after 48 h. However, this rate did not change after 72 h. A dose-dependent effect was not observed. The most effective MEs were those of *Dictyota cilliolata* (MEDC) and *Dictyota menstrualis* (MEDM). MEDC presented a time-dependent effect and achieved a cell viability inhibition rate of around 50% after 72 h of experiment (at 0.2 mg/mL). MEDM presented a dose-dependent and time-dependent effect, reaching its maximum inhibition (approximately 80%) with 0.2 mg/mL after 48 h exposure. 

In another study, Vinayak and colleagues [16] showed that ME (0.1 mg/mL) of *Spatoglossum asperum*, *Spatoglossum variabile* and *Sargassum marginatum* exhibited inhibition rates of around 82%, 80% and 40%, respectively, against EAT cells. In addition, these MEs did not affect the viability of normal cells. These results indicate that MEs of these two genera may be more effective against breast cancer cell lines. It is intended in the future to evaluate the effect of *S. schröederi* and *S. filipendula* extracts against breast tumor cells. 

In recent decades, representative anticancer substances extracted from seaweed include water soluble polysaccharides such as sulfated polysaccharides [18], and secondary metabolites [19], such as flavonoids and fluorotaninns. Thomas and Kim, for example, affirm that the antiproliferative activity in seaweed depends on the total polyphenol content [20]. However, despite these finds, relatively little is known about the chemical identity of the bioactivity compounds of these seaweed MEs and to date, scientific documentation of their bioactivity compounds are rare or non-existent. Therefore, we quantified the levels of sugar and phenolic compounds of ME and posteriorly, we have established a correlation between the amount of these compounds in the ME and the cytotoxic activity of these extracts. Analyses of the correlation coefficients between ME brown algae and green algae showed a positive correlation between inhibition of HeLa cell viability and content of both sugars and polyphenols. However, these correlations ranged from weak to moderate (Table 1). It is possible that the observed inhibition of cell viability is the result of combined effect of these two classes of compounds. Similar correlations were not made with red seaweed because we only had MEs from two species.

**Table 1:** Correlation coefficients between the amount of phenolic compounds or sugar of extracts and their cytotoxicity of each class of seaweeds.

Seaweeds	Sugar	Phenolic Compounds
Green	0.5161	0.3462
Brown	0.4321	0.3712

Among all seaweed extracts evaluated in this study the cytotoxic effects of MEDM and MEDC were the most evident. Comparing the cytotoxic effect of these extracts with those presented by other research groups, brown seaweed extracts like *Laminaria setchellii*, *Macrocystis integrifólia* and *Nereocystis leutkeana* showed no more than 20% of inhibition in HeLa cell viability, even using a higher concentration than in our case (0.5 mg/mL) [21].

**Figure 2:**
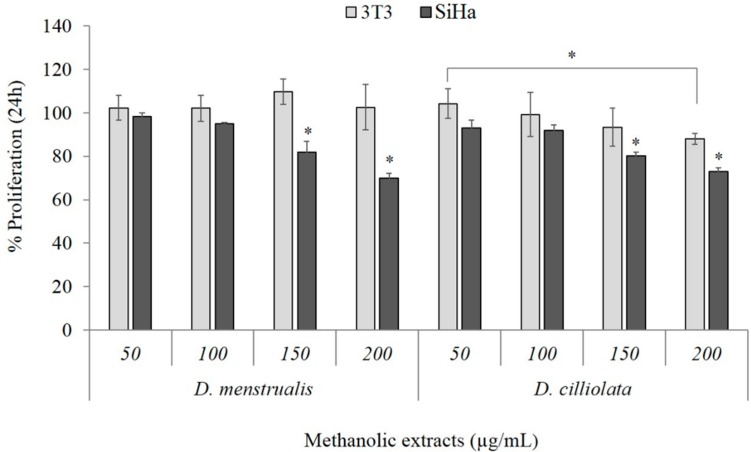
Effect of MEDM and MEDC on 3T3 and SiHa cells viability after 24 h of incubation. Data are expressed as mean ± standard deviation. * Indicates significant difference (*p* < 0.01) among the different concentrations of ME.

Subsequent assays were performed on cells with MEDC and MEDM because these two extracts were the ones that presented themselves as the most potent cytotoxic agents against HeLa cells. Thus, in order to identify whether the effect of the MEDM and MEDC is cell dependent, we evaluated its effect against squamous cell carcinoma (SiHa) cells, another human cervix tumor cell line. In addition, the effect of MEDM and MEDC against a non-tumor cell line, for instance fibroblasts 3T3, was also evaluated. Under all tested conditions (from 50 to 200 µg/mL for 24 h) cell viability rate of the 3T3 cells was not affected by the presence of MEDM (Figure 2), whereas MEDC only inhibits 3T3 proliferation (~20%) at higher concentration (200 µg/mL). On the other hand, both MEs affected the proliferation of SiHa cells (Figure 2), which however, were more resistant than the Hela cells.

HeLa and SiHa cells are human papillomavirus (HPV) positive. However, the kind of HPV virus in each cell are different, HeLa cells possess HPV 18, whereas HPV 16 is found in SiHa cells [22], which means they respond differently when exposed to same compound. That is why HeLa cells were more susceptible to the ME antiproliferative action than SiHa cells. Several studies showed similar data. For example, 3,3-diindolylmethane showed a stronger inhibitory effect on SiHa cells than HeLa cells [23]. On the contrary, cisplatin, a widely-used antitumor drug, was more effective on HeLa cells compared to SiHa cells [24].

**Figure 3:**
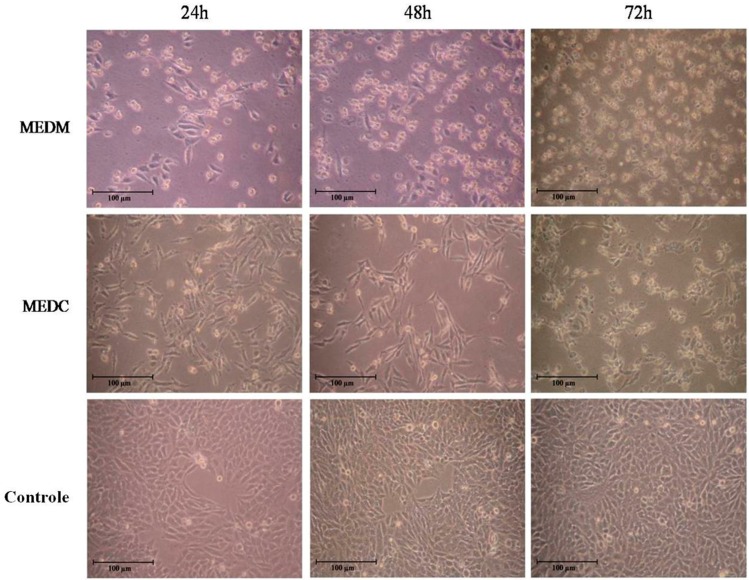
Morphological changes of HeLa cells after treatment with MEDM and MEDC for different times. HeLa cells were treated with extract (0.2 mg/mL) for 24 h. After incubation, the cells were examined under light microscopy. The data are a representative example for duplicate tests. Magnification ×100.

Figure 3 shows the morphological appearance of HeLa cells. The untreated HeLa cells exhibited typical growth patterns and normal morphology at all times analyzed. On the other hand, the MEDC and MEDM-treated cells showed a distinct morphological change characterized by shrinkage (reduced cell-to-cell contacts) and nuclear condensation. In addition, the frequency of round cells (probably dead cells) increased with increasing time of exposure to the MEDM and MEDC.

### 2.2. Changes in Nuclear Morphology

**Figure 4:**
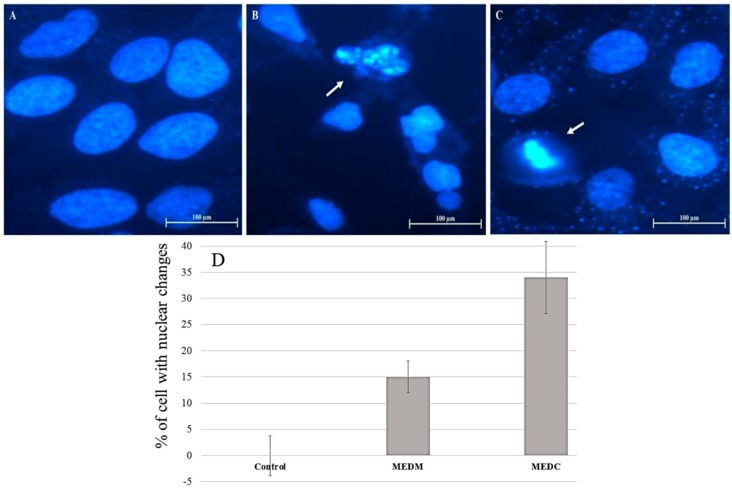
Morphological changes of HeLa cells after treatment with ME for 24 h followed by DAPI staining. (**A**) Fluorescence microscope photographs of untreated cells; (**B**) cells treated with 0.2 mg/mL MEDM and (**C**) cells treated with 0.2 mg/mL MEDC; (**D**) DAPI staining quantification. Arrows indicate apoptotic bodies of nuclear fragmentation and chromatin condensation (B and C, respectively). Magnification ×400.

Two of the earliest classic ultrastructural changes detectable in apoptosis are formation of uniformly dense masses of chromatin distributed against the nuclear envelope and persistence of a nucleolar structure until the very late stages [25]. In order to determine whether the cytotoxic effect of MEDC and MEDM was due to apoptosis, HeLa cells were treated with extracts for 24 h at 0.2 mg/mL concentration, and nuclear DAPI staining was performed. As shown in Figure 4, nuclei with condensed chromatin and apoptotic bodies of different sizes containing well-preserved but compacted cytoplasmic organelles and/or nuclear fragments, which are the typical characteristics of apoptosis [25], were observed in treated HeLa cells. The cells were counted and the percentage of cell with chromatin condensation or nuclear fragmentation in each image (see the Experimental Section) was used to produce Figure 4D. The findings showed that the MEDC extract induces a greater quantity of alterations in nuclear morphology compared to the MEDM extract, which confirms the data from the MTT assay.

### 2.3. MEDC and MEDM Effect for Labeling Cells with Propidium Iodide and Annexin V

Annexin V and propidium iodide (PI) are two labels used in order to differentiate cells undergoing apoptosis and necrosis. Generally, cells labeled with annexin V indicate initial apoptosis, cells labeled with PI are indicative of necrosis, and cells positive for annexin and PI are indicative of late apoptosis.

**Figure 5:**
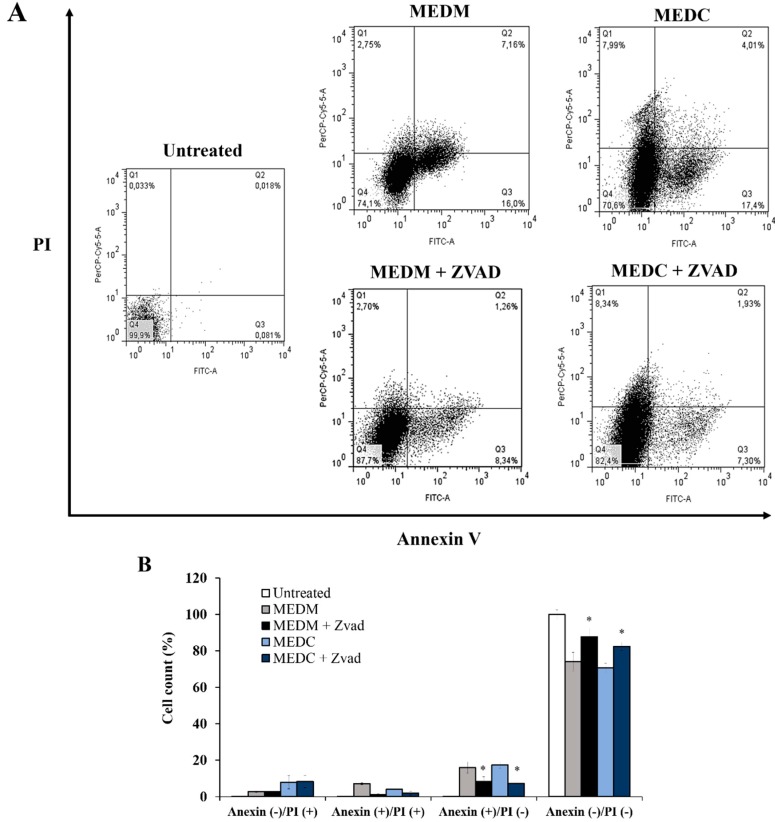
Flow cytometry analysis of HeLa cells after exposure toMEDC and MEDM. (**A**) Dot plots display the apoptotic death of HeLa cells treated with 0.1 mg/mL of ME and each extract with ZVAD treatment; (**B**) Each bar represents mean ± SD of triplicate experiments. The significance was determined by Turkey *t*-test (* *p* < 0.01 ME *vs.* ME treated with ZVAD). Annexin−/PI− (Q4), viable cells; Annexin+/PI− (Q3), cells undergoing apoptosis; Annexin+/PI+ (Q2), cells that are in end-stage apoptosis or are already dead; Annexin−/PI+ (Q1), cells that are in necrosis. One representative FACS assay of three independent experiments is presented.

Therefore, HeLa cells were incubated with MEDC and MEDM (0.1 mg/mL) for 24 h, and subsequently marked with PI and annexin V. It is worth noting that when using 0.2 mg/mL of extract, the number of cells after 24 incubation was very low which hampered the analyses. The data obtained are shown in Figure 5. Both MEDM and MEDC reduced the number of viable cells from 99% to ~70% in comparison to the control group. However, it can also be observed that the treatment with MEDM increased the percentage of annexin-positive (annexin V-FITC+/PI−) cells (16%) suggesting early apoptosis. The treatment of cells with MEDC also promoted an increase of 17% in the percentage of annexin-positive (annexin V-FITC+/PI) cells over the control group. However, differently from MEDM, treatment with MEDC also increased the percentage of PI-positive cells (7.99%) (annexin V-FITC−/PI+), that suggests the presence of cells in the initial necrotic stage as well as apoptosis. These data corroborate the results of nuclei morphological analysis that showed a greater number of nuclear changes (mainly apoptotic bodies) in the cells treated with MEDC.

In order to confirm the role of caspases in the MEDC and MEDM-induced apoptosis, HeLa cells were incubated with ZVAD-FMK. As can be seen in Figure 5B, in the presence of this pan-caspase inhibitor, the percentage of cells positive for annexin (annexin V-FITC+/PI−) decreased from 17% to 7.3% when MEDC was used and from 16% to 8.3% when the cells were incubated with MEDM. These data suggested that the main mechanism of action of MEDC and MEDM-induced apoptosis is caspase dependent. However, other cell death-inducing pathways are also being activated in the presence of MEDC and MEDM, since even in the presence of ZVAD the cytotoxic effect of MEDC and MEDM was not completely abolished.

### 2.4. MEDC and MEDM Treatment-Induced Apoptosis Require Activation of Caspases in HeLa Cells

For execution of apoptosis, activation of a family of caspases is necessary. Procaspase-9 as initiator is activated by self-processing and cleaves downstream procaspase-3 to active the dimeric form of caspase-3 to execute apoptosis.

**Figure 6:**
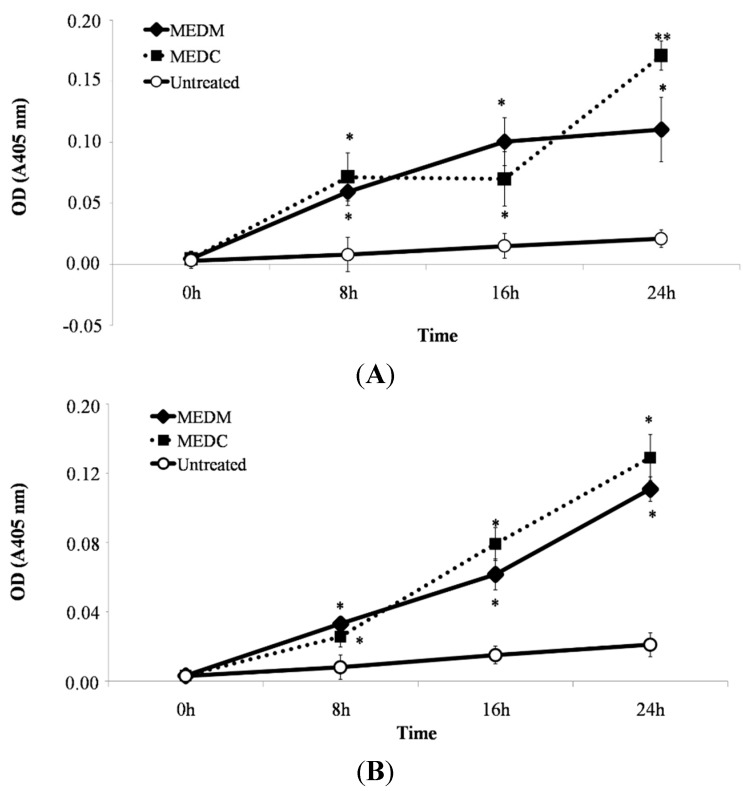
Effect of MEDM and MEDC on caspase-3 (**A**) and caspase-9 (**B**) activities in HeLa cells. The significance was determined by Turkey t-test (* *p* < 0.01 *vs.* negative control; ** *p* < 0.01 *vs.* ME).

To verify whether MEDC and MEDM induces activation of these caspases (9 and 3), treated HeLa cells were analysed by the spectrophotometric assay based on detection of the chromophore *p*-nitroanilide (pNA) after cleavage from the labeled substrate DEVD-pNA and LEHD-pNA, for caspase-3 and -9, respectively. These data demonstrate that activation of caspase-3 and 9-like activity is an essential event for MEDC and MEDM-induced apoptosis (Figure 6).

Two pathways are known to mediate anti-proliferative drug-induced apoptosis, death receptor-dependent (extrinsic) and mitochondrial-dependent (intrinsic) pathways. In mitochondria, caspase-9 was activated to form an apoptosome complex with Apaf-1 for subsequent activation of caspase-3 for apoptosis execution [26]. The finding of apoptosis, accompanied by increases in caspase-3 and caspase-9 activity during HeLa cell apoptosis, strongly indicate that apoptosis induced by MEDC and MEDM occurred via mitochondria-dependent signal pathway. Activation of caspase-9 and caspase-3 appears as the main antitumoral mechanism of various sulfated polysaccharides of brown marine macroalgae [27,28]. However, other means of inducing cell death that would produce the effects of MEDC and MEDM cannot yet be discarded. For example, sulfated polysaccharides from *Sargassum filipendula* induced apoptosis in HeLa promoted by the release of mitochondrial AIF into the cytoplasm (apoptosis inducing factor, independent of caspases) [29]. The analysis of the aforementioned data suggests that the mechanism of apoptosis induction by MEDC and MEDM s is very complex and further work will be dedicated to identifying the main cell proteins involved in the mechanism of action of MEDC and MEDM-induced apoptosis.

### 2.5. Cell Cycle Arrest

Cell cycle checkpoints ensure the maintenance of genomic integrity by protecting dividing cells from the potentially fatal consequences of DNA damage. The detection of DNA damage relies on a cascade of enzymes, conveying the signals generated by different genotoxic stresses that block key cell cycle transitions until DNA repair has occurred [30]. In the case of irreparable damage, the cells may be forced to withdraw definitively from the cell cycle or die by apoptosis so that cells do not replicate or segregate chromosomes bearing unrepaired lesions. Defects in the DNA damage checkpoint and/or the related cell cycle regulation network could contribute to the development of diverse types of mutations or chromosome rearrangements and promote tumorigenesis [31].

**Figure 7:**
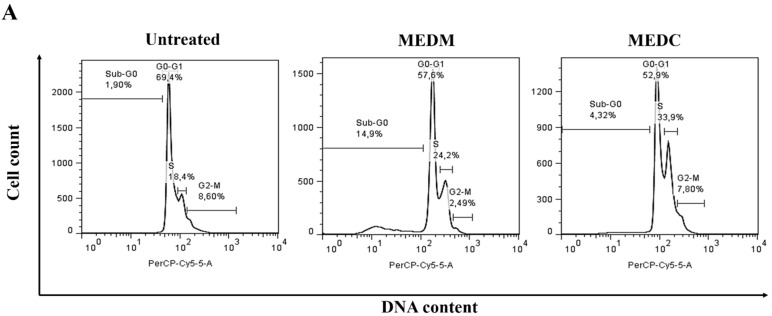

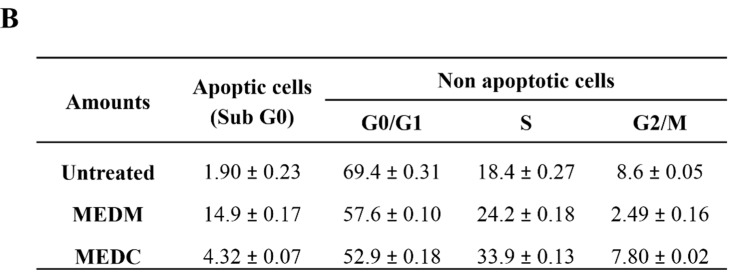
Cell cycle analysis of HeLa cells at 24 h. After treatment with MEDM and MEDC, cells were collected and stained with PI, and then flow cytometry analysis was performed. (**A**) Representation of the dot plots obtained from flow cytometry; (**B**) Table summarizes the mean of values obtained in three determinations ± standard deviation.

To determine whether MEDC and MEDM treatment of cells resulted in an alteration of cell cycle progression, the cell cycle patterns of the HeLa cells were examined. Representative blots are shown in Figure 7A and the quantification results are provided in Figure 7B.

Apoptosis was confirmed by the flow cytometry analysis, with the sub-G1 population (indicating apoptotic cells). Although the G1populationhas decreased along with an increase in sub-G1, the other phases of the cycle did not change significantly after exposure to MEDM. These results suggest that MEDM induces apoptosis in HeLa cells without inducing cell cycle arrest.

HeLa cells after exposure to MEDC had marked accumulation in the S and sub-G1 phases of the cell cycle when compared with the untreated control cells. The S phase of the cell division cycle represents the period in which cells replicate their DNA. If DNA replication is blocked by inhibitors or if the template is damaged by some factors, signals are generated that can induce cell-cycle arrest or apoptosis [32]. Therefore, we suggest that the growth inhibitory effect of MEDC in HeLa cells was result of a block during this S phase beyond its ability to induce apoptosis.

## 3. Experimental Section

### 3.1. Materials

4,6-Diamidino-2-phenylindole (DAPI), and 3-(4,5-dimethylthiazol-2-yl)-2-5-diphenyltetrazolium bromide (MTT), were purchased from Sigma Chemical Company (St. Louis, MO, USA). Cell culture medium components (Dulbecco’s Modified Eagle Medium-DMEM), trypsin and newborn calf serum (FCS) were obtained from Cultilab (Campinas, SP, Brazil). L-Glutamine, sodium bicarbonate, sodium pyruvate and phosphate buffered saline (PBS) were purchased from Invitrogen Corporation (Burlington, ON, Canada). All other solvents and chemicals were of analytical grade. Stock solution of ME (20 mg/mL) prepared by dissolving in 50% methanol was diluted with Dulbecco’s modified Eagles medium (DMEM) prior to use to obtain the desired concentration. The major final concentration of methanol used for treatment was 0.5% (*v*/*v*).

### 3.2. Preparation of the Seaweed Extracts

Thirteen species of seaweeds were collected along the coast of Natal, Brazil. The Phaeophyta: *Dictyopteris delicatula* (J.V. Lamouroux), *Dictyota ciliolate* (Sond. ex Kütz), *Dictyota menstrualis* (Hoyt) (Schnetter, Hörnig & Weber-Peukert), *Dictyota mertensii* (Martius) (Kützing), *Spatoglossum schroederi* (C. Agardh) (Kützing) and *Sargassumfilipendula* (C. Agardh). The Rodophyta: *Acanthophora spicifera* (Vahl) (Børgesen), *Botryocladia occidentalis* (Børgesen) (Kylin) and the Clorophyta *Caulerpa cupressoides* var. *flabellate* (Børgesen); *Caulerpa sertularioides* (S. G Gmelin) (M Howe); *Caulerpa prolifera* (Förssk.) (J. V. Lamour); *Caulerpa racemosa* (Försskall) (J. Agardh) var. *occidentalis* (J. Agardh) (Börgensen); *Codium isthmocladum* (Vickers). They were identified by Dr. Hugo A. O. Rocha, UFRN, Brazil. The seaweeds were washed with water, cut into small pieces and air-dried at 50 °C. One gram of dried sample was suspended in 10 mL of methanol by shaking for 24 h in the dark. The solution was filtered and evaporated to dryness under reduced pressure. The dried powder was dissolved in 50% distilled water and methanol (*v*/*v*) and stored at −20 °C until use.

### 3.3. Chemical Analyses

The quantification of sugar in the extracts was performed determination of sugars by the method phenol/sulfuric acid, using galactose or fucose as standard [33]. Phenolic compounds were quantified by the colorimetric method of Folin-Ciocalteu reagent using gallic acid as standard [34].

### 3.4. Cell Culture and Maintenance

HeLa cervical adenocarcinoma cells (ATCC CCL-2) were donated by Dr Silvia R. Batistuzzo Medeiros (Department of Genetics and Molecular Biology, UFRN, Natal, RN, Brazil) and embryo fibroblast 3T3 (ATCC CCL-164) was donated by Dr Carmen Ferreira (Department of Biochemistry, UNICAMP, Campinas, SP, Brazil). SiHa human squamous cell carcinoma (ATCC HTB-35) was donated by Dr Silvana Maria Zucolotto Langassner (Department of Pharmacy, UFRN). The cells were grown as previously described by Almeida-Lima *et al.* [35]. The cells were grown in DMEM supplemented with 10% FBS (Sigma) and antibiotics (100 U/mL of penicillin and 100 μg/mL of streptomycin). Cells were maintained as monolayer cultures in a humidified atmosphere of 5% CO_2_ at 37 °C. HeLa cells were seeded at a density of 5 × 10^6^ for 75 cm^3^ flasks.

### 3.5. Cytotoxicity Activity

Cytotoxicity activity of ME was determined using the MTT assay as previously described by Almeida-Lima *et al.* [35]. The MTT assay is based on the reduction of MTT by mitochondrial dehydrogenases of viable cells to a purple formazan product [36]. Briefly, HeLa cells were seeded into 96-well plates at a density of 5 × 10^3^ cells/well and allowed to attach overnight in 100 μL medium FCS free incubated at 37 °C, 5% CO_2_. The medium was then removed and 100 μL of medium/10% FCS plus ME diluted to a final concentration of 0.05, 0.1, 0.15 or 0.2 mg/mL. Cells were grown under these conditions for 24 h, 48 h and 72 h at 37 °C at 5% CO_2_. After incubation, traces of ME were removed by washing the cells twice with 200 μL PBS and applying MTT (1 mg/mL) dissolved in 100 μL of fresh medium to determine the effects of the ME on cell viability. Cells were then incubated for 4 h at 37 °C, 5% CO_2_. The MTT-formazan product dissolved in 100 μL of ethanol was estimated by measuring the absorbance at 570 nm in a Multiskan Ascent Microplate Reader (ThermoLabsystems, Franklin, MA, USA). Proliferation is presented as a percentage of cell proliferation under no treatment conditions. For the 3T3 cell line this same procedure was performed, but the cells were incubated for a period of 24 h only with MEDC and MEDM at 0.2 mg/mL concentration. All concentrations were tested in triplicate and the experiment was repeated at least three times.

### 3.6. Nuclear Morphology

The changes in the nuclear morphology of cells following exposure to ME (0.2 mg/mL) were examined using the DNA-specific fluorochrome DAPI. Cells were plated at a density of 3 × 10^4^ cells/well in a 24-well plate and after 24 h of incubation, cells were treated with MEDC and MEDM for 24 h. Cells were washed with PBS and fixed with 4% paraformaldehyde in PBS for 30 min at room temperature. After washing twice with PBS, cells were maintained in PBS containing 0.1% Triton X-100 at room temperature for 30 min. Fixed cells were washed with PBS and stained with DAPI (1 μg/mL) solution for 30 min at room temperature. Nuclear morphology of apoptotic cells with condensed/fragmented nuclei was examined under a fluorescent microscope (TE-Eclipse 300, Nikon, Melville, NY, USA). Data presented are representative of those obtained in at least three independent experiments done in duplicate.

Chromatin condensation and nuclear fragmentation were the criteria used to demonstrate apoptosis. DAPI staining was also used to quantify apoptotic cell. Images were captured from ten different fields and about 100 cells were counted for each field on UV microscopy and analyzed using the NIS-Elements AR analysis software version 4.00.03 (Nikon Instruments Inc., 2011). Counting was done in three independent experiments for each sample.

### 3.7. Annexin V-FITC/PI Double Staining and Analysis by Flow Citometry

In order to evaluate the effects of extracts on cell death, a FITC/Annexin V Apoptosis Kit was used, with Dead Cell Annexin FITC and propidium iodide (PI), for Flow Cytometry (Invitrogen). Cells were grown in 6-well plates until they reached confluence of 2 × 10^5^ cells/mL and were then stimulated to enter G0 in a medium without serum for 24 h. Next, cells were to exit G0 by adding DMEM supplemented with 10% FCS, in the presence of MEDC or MEDM (0.1 mg/mL). A negative control was prepared without the presence of MEDC or MEDM. The cell effect of MEDC or MEDM incubated with pan-caspase inhibitor ZVAD-FMK (carbobenzoxyvalyl-alanyl-aspartyl-[*O*-methyl]-fluoromethylketone) was also tested. After 24 h, HeLa cells were trypsinized, collected and washed with cold PBS. The supernatant was discarded and cells were resuspended in 200 µL of 1× Binding Buffer. Five microlitres of Annexin V-FITC and 1 µL of PI solution 100 µg/mL was added in 100 µL of cell suspension. Cells were incubated for 15 min at room temperature and kept under light protection. After the incubation period, 400 µL of binding buffer for annexin V 1× was added and cells were analyzed by flow cytometry (FASCANTO II flow cytometer, BD Biosciences, São Paulo, SP, Brazil), measuring fluorescence emission at 530–575 nm for annexin V and 630/22 nm for PI. A total of 10.000 events were acquired. FlowJo software v. 9.3.2 (Tree Star, Inc., Ashland, OR, USA) was used for data analysis as described in Costa *et al.* [29].

### 3.8. Caspase-3 and -9 Activity Assay

HeLa cells were placed in a Petri dish (1 × 10^6^ cells/mL), and then stimulated to enter G0 in a medium without serum for 24 h. Next, cells were to exit G0 by adding DMEM supplemented with 10% FCS, in the presence of MEDC or MEDM (0.2 mg/mL), washed after 8, 16 and 24 h in ice-cold PBS and scraped into 200 mL lysis buffer [50 mM Tris-HCl (pH 7.4), 1% Tween 20, 0.25% sodium deoxycholate, 150 mM NaCl, 1 mM EDTA, 1 mM Na_3_VO_4_, 1 mMNaF], and protease inhibitors [1 mg/mL aprotinin, 10 mg/mL leupeptin and 1 mM 4-(2-aminoethyl) benzenesulfonyl fluoride] for 2 h in ice. The same conditions were used for untreated cells in the 0, 8, 16 and 24 h. Protein extracts were cleared by centrifugation and protein concentrations were determined using Bradford reagent [37] with bovine serum albumin as standard. *In vitro* caspase-3 and -9 protease activity was measured using a caspase activation kit according to the manufacturer’s protocol (Invitrogen). For this, 50 µL of cell lysate was mixed with 50 µL of 2× reaction buffer [containing 10 µL of 1 M dithiothreitol and 5 µL of 4 mM synthetic tetrapeptide Asp-Glue-Val (for caspase 3) or Leu-Glu-His-Asp (for caspase 9) conjugated top-nitroanilide (pNA)] in 96-well plate, after which the mixture was incubated for 2 h at 37 °C in the dark. Active caspase cleaves the peptide and releases the chromophore pNA that can be detected spectrophotometrically at a wavelength of 405 nm. Theoretically, the apoptotic cell lysates containing active tested caspases should yield a considerable emission compared with the non-apoptotic cell lysates. Data presented are representative of those obtained in at least three independent experiments done in duplicate.

### 3.9. Cell Cycle Analysis

HeLa cells were placed in a 6-well plate (2 × 10^5^ cells/mL) and were then stimulated to enter G0 in a medium without serum for 24 h. Next, cells were to exit G0 by adding DMEM supplemented with 10% FCS, in the presence of MEDC or MEDM (0.2 mg/mL). After 24 h, the cells were harvested washed twice with cold PBS and centrifuged, cell pellets were fixed in 2% paraformaldehyde for 30 min. After washing twice with PBS to remove paraformaldehyde excess, cells were incubated in PBS containing 0.01% saponin and RNase (4 mg/mL) at 37 °C for at least 45 min. Subsequently, 2 μL of PI and 200 μL of PBS were added protected from light. The DNA content was analyzed using a FACSCalibur flow cytometer. A total of 30.000 events were acquired. For data analysis, was used FlowJo^®^ Analysis Software version 9.3.2. Data presented are representative of those obtained in at least three independent experiments done in duplicates.

### 3.10. Statistics Analysis

We used analysis of variance (ANOVA) and Turkey t-test. Differences with *p* < 0.01 between the values are considered statistically significant. Statistical analysis and the Pearson correlation coefficient (ρ) were performed using GraphPadInStat^®^ Software version 4.0 (GraphPad software, San Diego, CA, USA).

## 4. Conclusions

In synthesis, cells exposed to MEDM and MEDC exhibit the morphological and biochemical changes that characterize apoptosis as shown by loss of cell viability (Figure 1 and Figure 2), chromatin condensation (Figure 4), phosphatidylserine externalization (Figure 5), and sub-G1 phase accumulation (Figure 6). Moreover, activation of caspases 3 and 9 suggests apoptosis involves a mitochondria-dependent route (Figure 7). However, other means of inducing cell death that would serve to the effects of MEDC and MEDM cannot yet be discarded.

Together these findings showed that the methanolic extracts from brown seaweeds *D. mentrualis* and *D. cilliolata* may be potential adenocarcinoma cell agents. Moreover, these MEs did not change the viability of normal cells (Figure 3). The present investigation points to the necessity of deeper phytochemical and biological investigation, because these species are potentially interesting in yielding biologically active products. As a continuation of this work, the active compounds will be isolated and their mechanisms of action better elucidated.
